# The vascular disrupting agent combretastatin A-4 phosphate causes prolonged elevation of proteins involved in heme flux and function in resistant tumor cells

**DOI:** 10.18632/oncotarget.23734

**Published:** 2017-12-28

**Authors:** Sanchareeka Dey, Sharda Kumari, Sarada Preeta Kalainayakan, James Campbell, Poorva Ghosh, Heling Zhou, Keely E. FitzGerald, Maoping Li, Ralph P. Mason, Li Zhang, Li Liu

**Affiliations:** ^1^ Department of Biological Sciences, The University of Texas at Dallas, Richardson, TX, USA; ^2^ Department of Radiology, The University of Texas Southwestern Medical Center, Dallas, TX, USA; ^3^ Visiting Fellow from Department of Ultrasound, The First Affiliated Hospital of Chongqing Medical University, Chongqing, China

**Keywords:** combretastatin A-4 phosphate (CA4P), multispectral optoacoustic tomography (MSOT), vascular disrupting agent (VDA), heme, lung tumor

## Abstract

Vascular disrupting agents (VDAs) represent a promising class of anti-cancer drugs for solid tumor treatment. Here, we aim to better understand the mechanisms underlying tumor reccurrence and treatment resistance following the administration of a VDA, combretastatin A-4 phosphate (CA4P). Firstly, we used photoacoustic tomography to noninvasively map the effect of CA4P on blood oxygen levels throughout subcutaneous non-small cell lung cancer (NSCLC) tumors in mice. We found that the oxygenation of peripheral tumor vessels was significantly decreased at 1 and 3 hours post-CA4P treatment. The oxygenation of the tumor core reduced significantly at 1 and 3 hours, and reached anoxia after 24 hours. Secondly, we examined the effect of CA4P on the levels of proteins involved in heme flux and function, which are elevated in lung tumors. Using immunohistochemistry, we found that CA4P substantially enhanced the levels of enzymes involved in heme biosynthesis, uptake, and degradation, as well as oxygen-utilizing hemoproteins. Furthermore, measurements of markers of mitochondrial function suggest that CA4P did not diminish mitochondrial function in resistant tumor cells. These results suggest that elevated levels of heme flux and function contribute to tumor regrowth and treatment resistance post-VDA administration.

## INTRODUCTION

Tumors cannot grow beyond 2 mm^3^ without a vascular structure to supply tumor core cells with oxygen and nutrients [1]. As such, disruption of tumor vasculature is a promising therapeutic strategy for treating solid tumors. Vascular disrupting agents (VDAs) act on tumor endothelial cells and induce a vascular shutdown, leading to reduction in blood flow, ischemia, and cell death [2, 3]. Combretastatin A-4 phosphate (CA4P) is the leading VDA candidate and is currently in various clinical trials in combination with chemotherapy or radiation therapy [4, 5]. CA4P inhibits microtubule polymerization by binding to the colchicine-binding site of beta-tubulin [6, 7]. Previous studies have examined the effect of CA4P *in vivo* with various non-invasive imaging techniques, such as MRI, infrared spectroscopy, and positron emission tomography (PET) [8–19]. While VDA treatment causes acute ischemia leading to extensive central necrosis in many tumors, a peripheral rim of viable tumor cells remains, which allows re-growth and re-vascularization of the tumor [20–23]. Further understanding of the mode of CA4P action may help to improve the anti-tumor efficacy of VDAs, particularly through rational combination with additional therapy.

Lung cancer is the leading cause of cancer-related deaths in the US; about 85% of cases are classified as non-small cell lung cancer (NSCLC) [24, 25]. Despite the advent of targeted therapies and immunotherapies, an effective treatment or cure for lung cancer remains an unlikely outcome for most patients. Thus, we decided to use imaging and immunohistochemistry (IHC) techniques to examine how NSCLC tumors respond to VDAs. Photoacoustic tomography (PAT) is a non-invasive technique for structural, functional, and molecular imaging [26]. It has been used to monitor tissue hypoxia and to detect biodistribution of nanoparticles in murine models [27–29]. Multispectral approach Optoacoustic Tomography (MSOT) provides high spatial and temporal resolution *in vivo* imaging for characterizing tumor vasculature in small animals. It involves illuminating the sample with multiple wavelengths (680-900 nm) sequentially. Spectral unmixing is used to differentiate between numerous different molecular species simultaneously [30, 31]. The promise of this technique lies in its ability to detect the conversion of light absorbance to a sound wave that produces a favorable signal-to-noise ratio, resolution, and penetration depth [27]. The optical absorption in biological tissues can be due to endogenous molecules such as hemoglobin (oxygenated hemoglobin and deoxygenated hemoglobin) or exogenously administered contrasting agents. Prompted by a few previous studies of vascular disrupting agents using spectrally resolved photoacoustic imaging [32, 33], we have applied MSOT to explore the mode of action of CA4P on lung tumors.

In this report, we use MSOT in combination with bioluminescence imaging (BLI) to examine the effect of CA4P on tumor vasculature in human lung H1299-Luc xenograft tumors. We detected tumor hypoxia immediately after CA4P treatment and recovery of tumor oxygenation 24 hours after treatment. Further, we detected the effect of CA4P on the levels of a wide array of cellular proteins critical for tumor development and progression using IHC. Intriguingly, we found that CA4P induced prolonged elevation in proteins involved in heme biosynthesis, uptake, and degradation in resistant tumor cells. The levels of a putative heme sensor and chaperone, PGRMC1, as well as other hemoproteins, such as COX-2 and cytochrome c, were also increased. Notably, the level of mitochondrial fission protein Drp1, which promotes mitochondrial fission and fragmentation [34], was reduced in resistant tumor cells, suggesting that tumor cells are not apoptotic. Conversely, the level of mitofusion 2 (Mfn2), which promotes mitochondrial clustering and fusion [35], was increased in resistant tumor cells. Taken together, our imaging and IHC data strongly suggest that CA4P causes elevated heme flux and function in viable NSCLC tumor cells, both of which likely contribute to re-growth and re-vascularization of tumors after treatment. Our results provide novel insights into how to improve anti-tumor efficacy of VDAs.

## RESULTS

**Figure 1:**
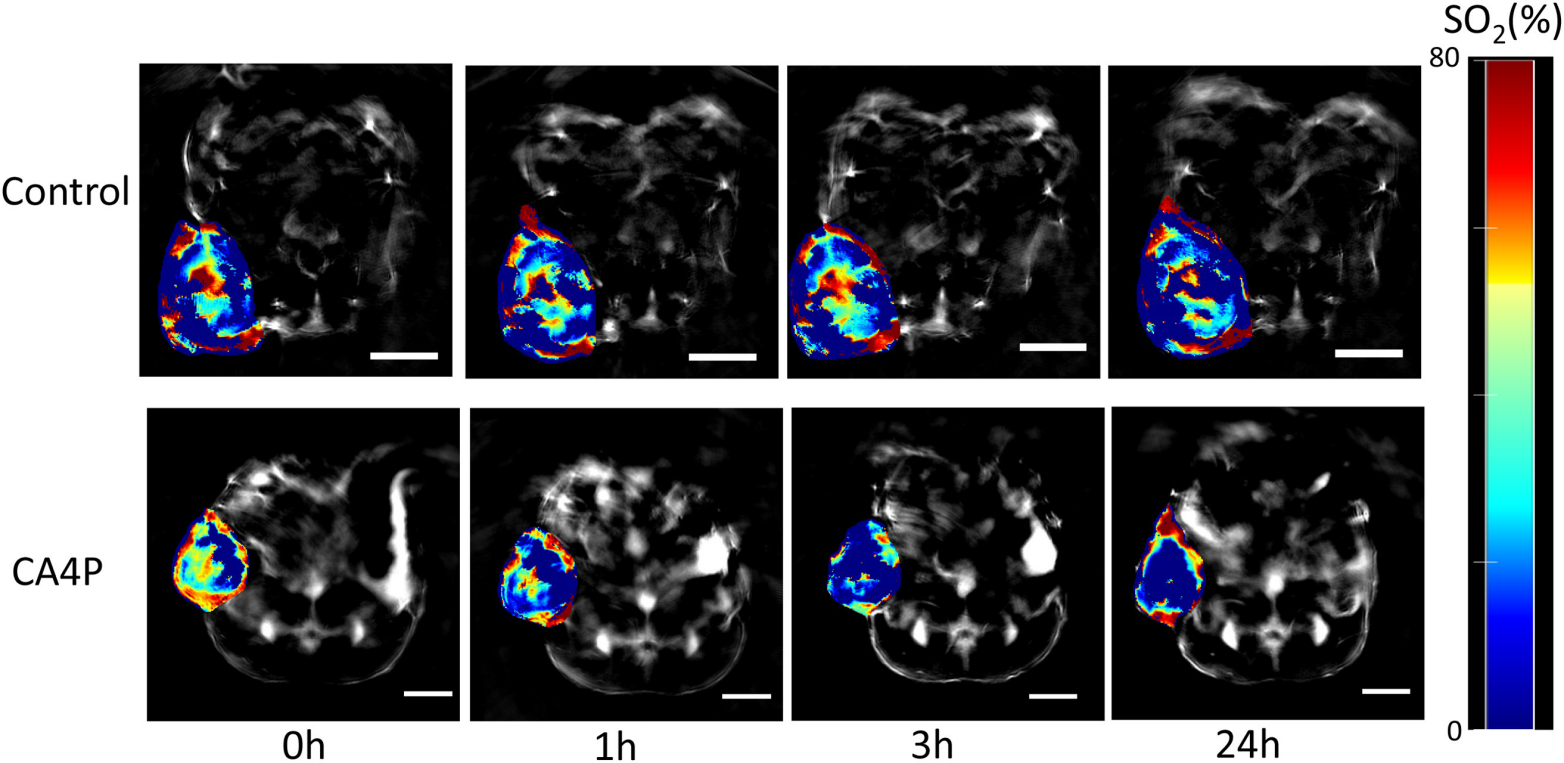
MSOT images of lung xenograft tumor at different time points (0h, 1h, 3h, and 24h) post-saline (control) or CA4P (120 mg/kg, IP) administration Red colour corresponds to higher concentration of oxyhemoglobin and blue colour represents either higher concentration of deoxyhemoglobin or an area where there is no blood present. Percentage of hemoglobin saturation (%SO_2_) was calculated as %SO_2_ = [HbO_2_/(HbO_2_+Hb)]^*^100. Paired Student’s t-test showed that the changes in %SO_2_ at 1h and 3h post-CA4P administration, compared to 0h, were statistically significant in tumor periphery (1h: p value <0.005, n=6; 3h: p value <0.05, n=4; 24h: not significant, n=4). The changes in %SO_2_ at 1h, 3h, and 24h post-CA4P administration, compared to 0 h, were statistically significant in tumor center (1h: p value <0.05, n=6; 3h: p value <0.05, n=4; 24h: p value <0.05, n=4). The variations in %SO_2_ in control tumors were of no statistical significance (n=4 for all time points), scale bar: 5 mm.

**Figure 2:**
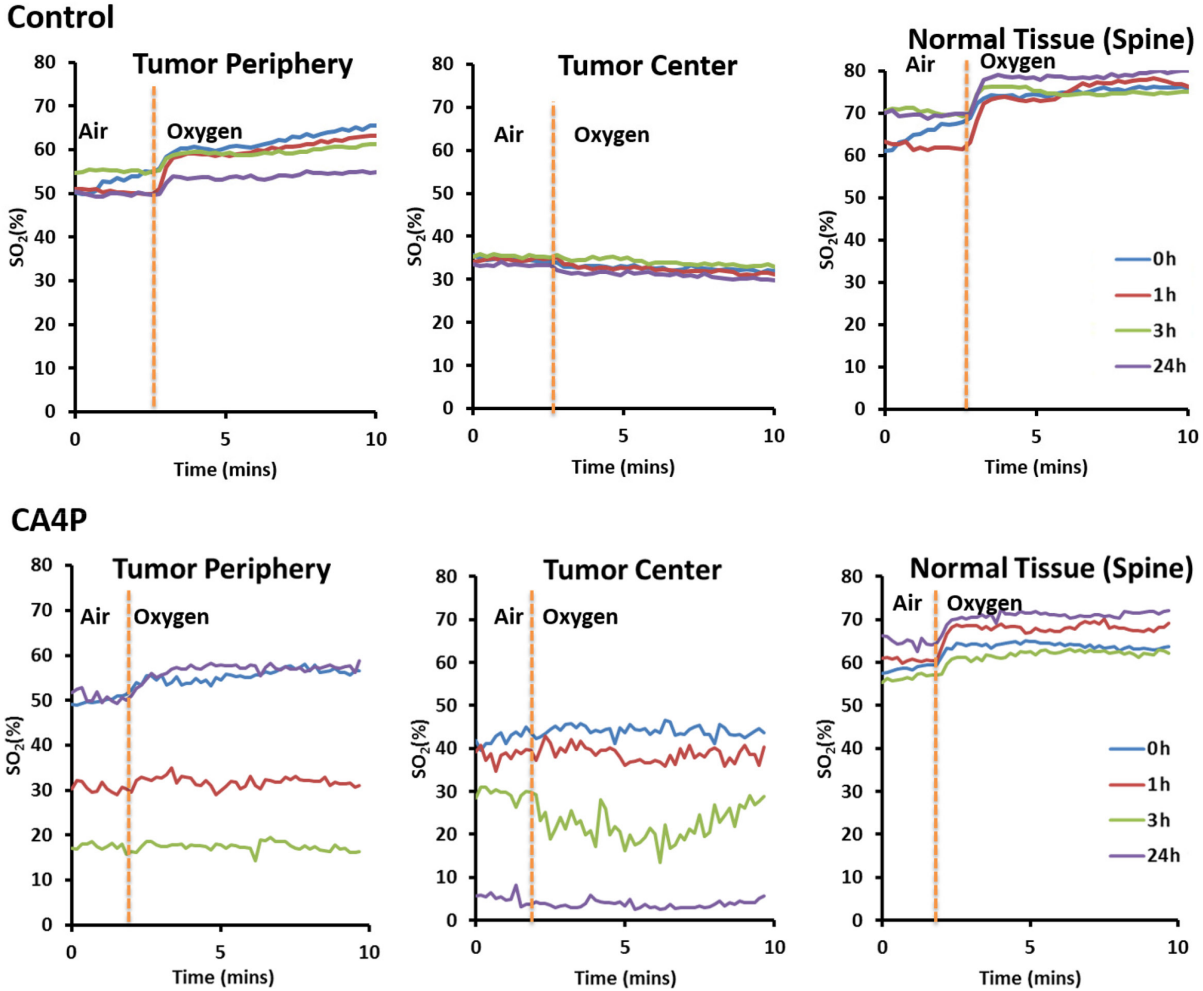
Graph showing change in blood oxygenation level at different time points (0h, 1h, 3h, and 24h) after administration of saline (control) or CA4P (120 mg/kg, IP) in different sections of tumor (tumor periphery and tumor center) compared to healthy tissue (spine) Percentage of hemoglobin saturation (%SO_2_) was calculated as %SO_2_ = [HbO_2_/(HbO_2_+Hb)]^*^100. Mice breathed air initially, and then the inspired gas was changed to oxygen.

Using MSOT, we found that CA4P caused a significant reduction in vascular oxygen saturation and hypoxia at 1 and 3 hours post-VDA treatment. The %SO_2_ of peripheral tumor vessels decreased from 49% at baseline to 30% (p value <0.005) and 18% (p value <0.05) at 1 and 3 hours, respectively (Figures 1 & 2). At the periphery of the tumor, %SO_2_ recovered partially after 24 hours. The %SO_2_ of the tumor core declined from 43% at baseline to 37% (p value <0.05) and 28% (p value < 0.05) at 1 and 3 hours respectively and continued to decrease to 0% (p value <0.005) at 24 hours (Figures 1 & 2). There was no change in %SO_2_ of normal tissue (spine). Similarly, in control mice, no significant change in %SO_2_ of tumor periphery, tumor center and spine were observed (Figures 1 & 2), although heterogeneity was observed in baseline tumor values.

**Figure 3:**
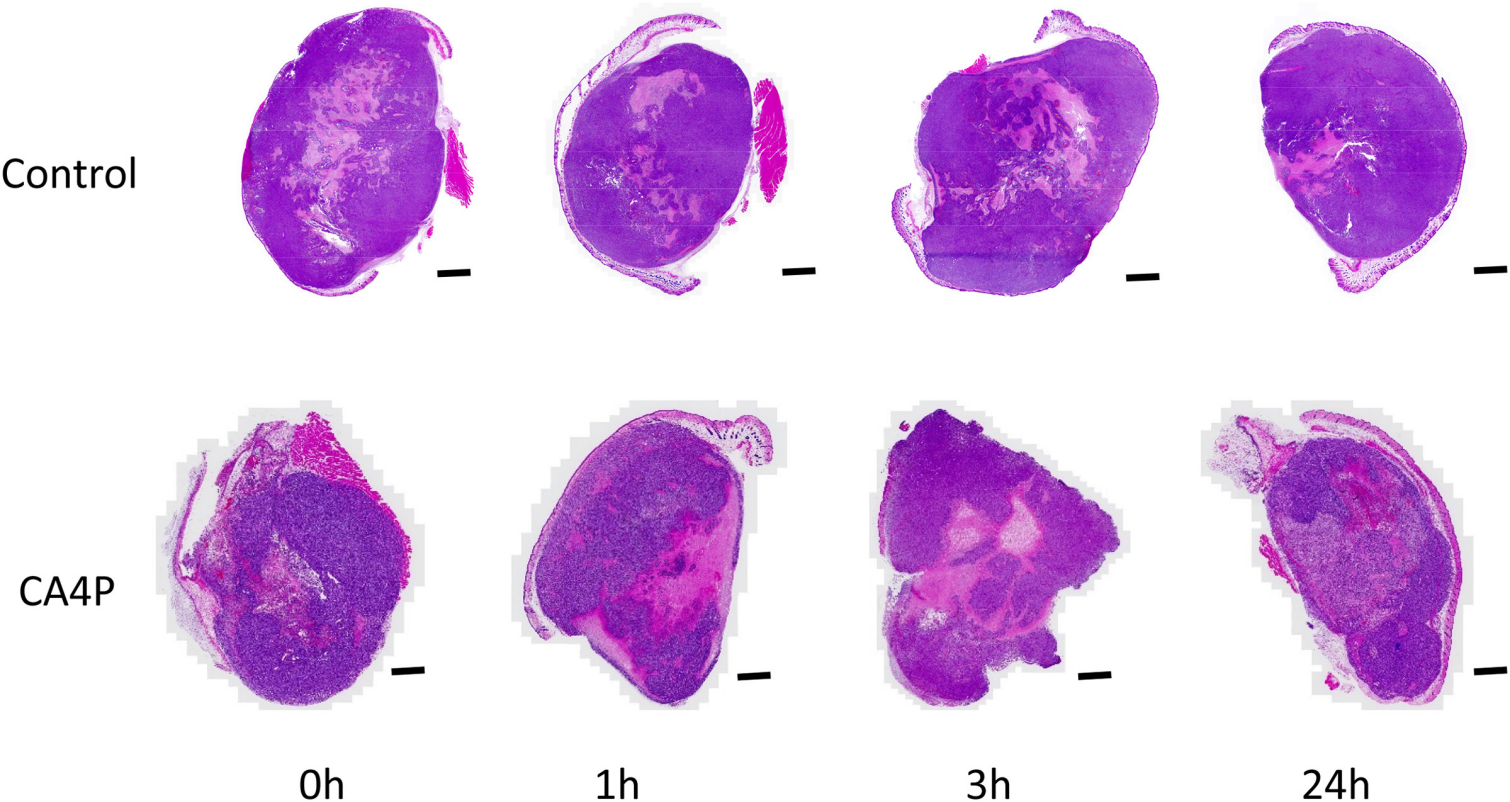
H&E staining image showing tumor response at different time points (0h, 1h, 3h, and 24h) after saline (control) or CA4P administration (120 mg/kg, IP), scale bar: 2 mm

BLI also detected reduced photon signal intensity at 3 hours after administration of CA4P (Supplementary Figure 1), indicating vascular shutdown. In contrast, signal intensity was observed to be highly consistent in saline-treated mice after 3 and 24 hours (Supplementary Figure 1). H & E staining indicated that there were large necrotic areas in the tumor center at both 3 and 24 hours post-CA4P administration (Figure 3). These results were consistent with previous studies examining the effects of VDAs on various tumors [36–40]. The necrotic areas at 3 and 24 hours post-CA4P administration were quantified and estimated to be approximately 29% and 46% of the entire tumor, respectively.

**Figure 4:**
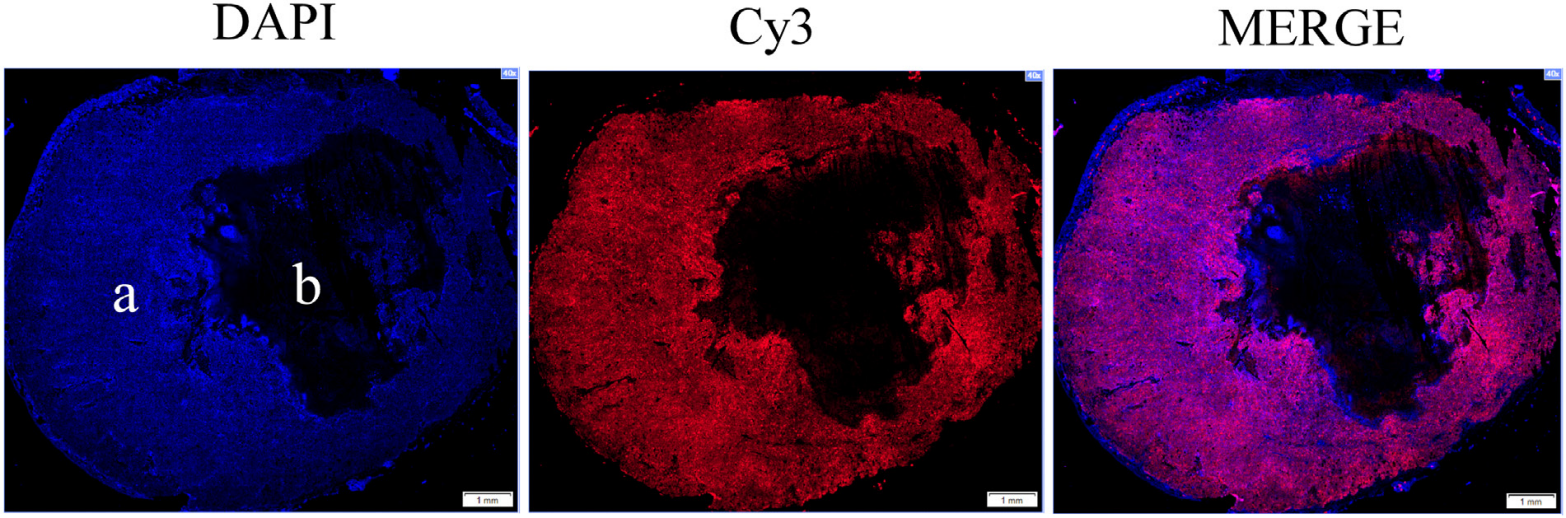
Nuclear marker DAPI was used to ensure that protein expression was detected only in areas with tumor cells **(a)** and not necrotic areas **(b)**. Left to right, 40X montage of paraffin sections of 3 hour CA4P treated subcutaneous xenograft tumors stained with anti-PGRMC1 antibody (red), DAPI for nuclei (blue), and merged image (red and blue). Scale bar, 1 mm.

**Figure 5:**
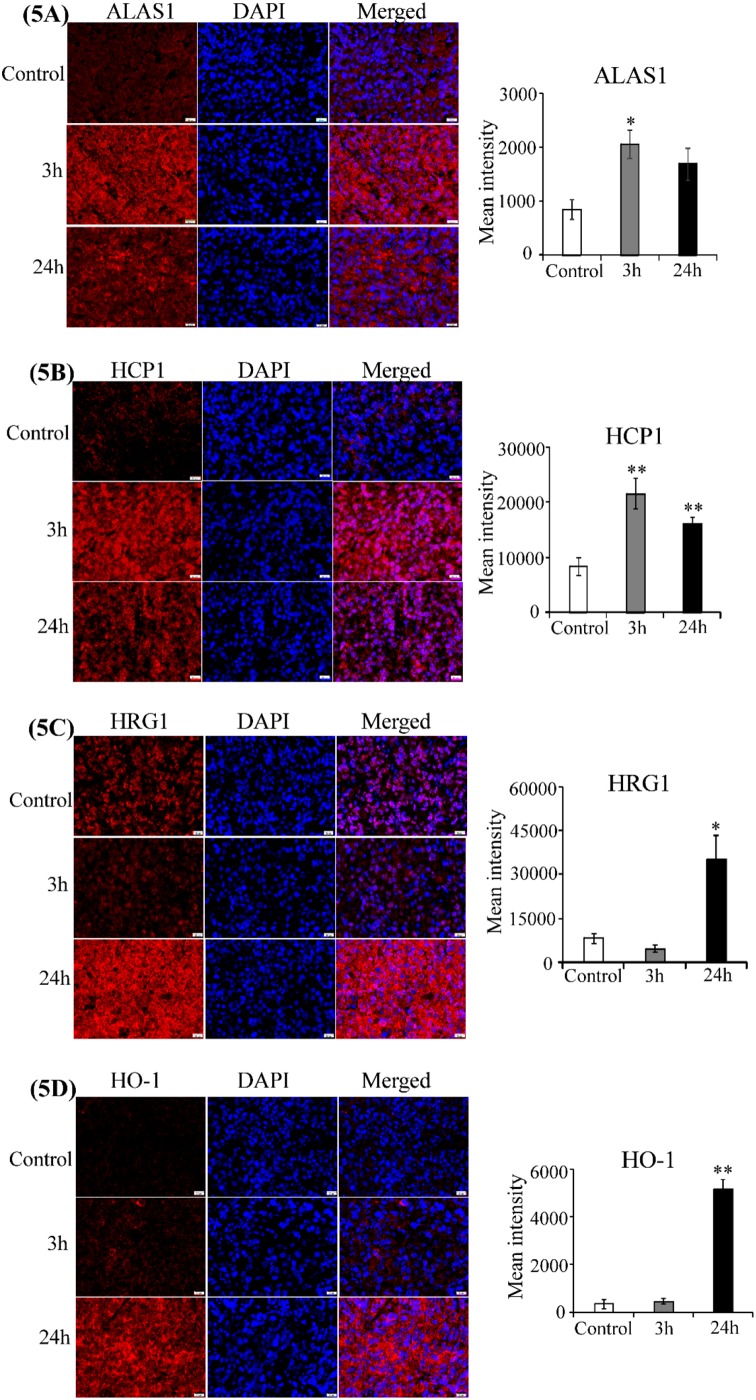
CA4P induces heme-related proteins Representative images of CA4P-treated, fluorescent immunohistochemically stained paraffin sections of subcutaneous xenograft tumors including untreated control (Row 1), 3 hours post-CA4P (Row 2), and 24 hours post-CA4P (Row 3). Bar graphs indicate the mean grey intensity of 10 ROIs quantified with cellSens dimension software (Olympus). Data are presented as mean ± SEM (n=10, ^*^p value < 0.05; ^**^p value < 0.005; scale bar, 20 μm). **(A)** ALAS1 is significantly elevated 3 hours post-treatment. Anti-ALAS1 antibody (red), DAPI for nuclei (blue), and merged image (red and blue). **(B)** HCP1 is significantly elevated 3 hours and 24 hours post-treatment. Anti-HCP1 antibody (red), DAPI for nuclei (blue), and merged image (red and blue). **(C)** HRG1 is significantly elevated 24 hours post-treatment. Anti-HRG1 antibody (red), DAPI for nuclei (blue), and merged image (red and blue). **(D)** HO-1 is significantly elevated 24 hours post-treatment. Anti-HO-1 antibody (red), DAPI for nuclei (blue), and merged image (red and blue).

To gain insights into the mechanisms underlying tumor recurrence and treatment resistance associated with a viable tumor rim after VDA treatment [20–23], we examined the effect of CA4P on the levels of cellular proteins critical for lung tumorigenesis. Notably, recent studies in the author’s laboratory have shown that proteins related to heme flux and function is elevated in NSCLC cells [41–43]. This elevation is critical for the tumorigenesis of NSCLC. We therefore examined the levels of proteins related to heme flux and function in CA4P-treated tumors with IHC. Firstly, to ensure that proteins were detected in areas with tumor cells, not necrotic areas, we used DAPI, a nuclear marker, to identify areas with intact tumor cells (Figure 4). Next, we detected the levels of the rate-limiting heme synthetic enzyme ALAS1; heme transport proteins HRG1 and HCP1, which allow heme uptake by tumor cells; and the heme degradation enzyme HO-1 [43]. Figure 5A shows that ALAS1 level was significantly elevated in tumor cells 3 hours after CA4P administration. Likewise, the level of HCP1 was significantly elevated at 3 and 24 hours post-treatment (Figure 5B), and the level of HRG1 was significantly elevated 24 hours post-treatment (Figure 5C). Notably, the level of HO-1 was highly elevated 24 hours after treatment (Figure 5D). Our data here show that VDA treatment increases the levels of enzymes involved in heme biosynthesis, uptake, and degradation in the resistant tumor cells.

**Figure 6:**
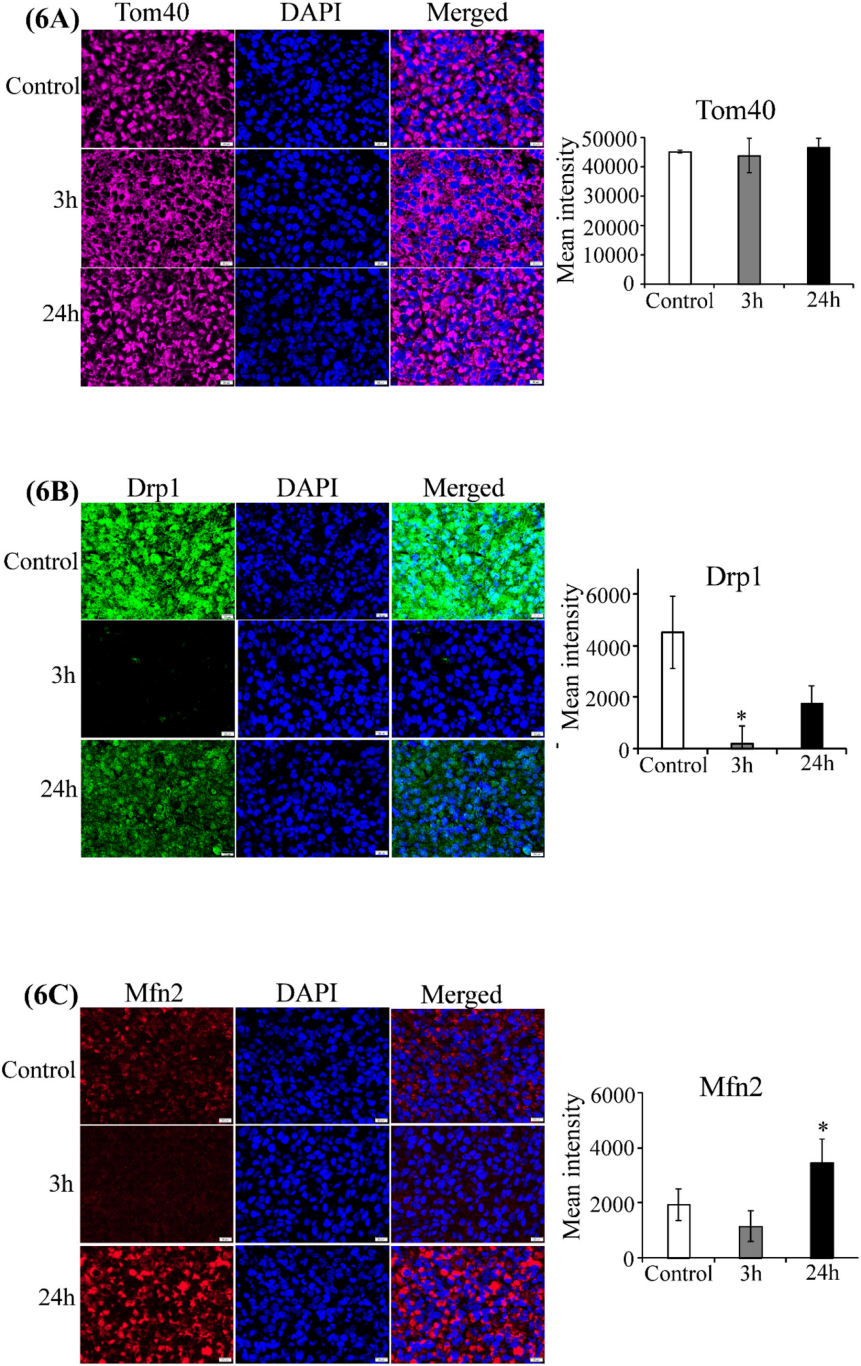
CA4P treatment does not disrupt mitochondrial function Representative images of CA4P-treated, fluorescent immunohistochemically stained paraffin sections of subcutaneous xenograft tumors including untreated control (Row 1), 3 hours post-CA4P (Row 2), and 24 hours post-CA4P (Row 3). Bar graphs indicate the quantification of mean grey intensity. Data are presented as mean ± SEM (n=10, ^*^p value < 0.05; ^**^p value < 0.005; scale bar, 20 μm). **(A)** Tom40 is unaffected by CA4P treatment. Anti-Tom40 antibody (cyan), DAPI for nuclei (blue), and merged image (cyan and blue). **(B)** Drp1 is reduced 3 and 24 hours post-treatment. Anti-Drp1 antibody (green), DAPI for nuclei (blue), and merged image (green and blue). **(C)** Mfn2 is enhanced at 24 hours post-treatment. Anti-Mfn2 antibody (red), DAPI for nuclei (blue), and merged image (red and blue).

Subsequently, we detected the effect of CA4P on two hemoproteins, COX-2 and cytochrome c. We found that the level of cytochrome c was significantly elevated 3 hours after treatment (Supplementary Figure 2A), although 24 hours after treatment, the cytochrome c level was not significantly different from untreated tumors. Strikingly, the level of COX-2 was significantly enhanced at both 3 and 24 hours post-treatment, with high elevation at 24 hours post-treatment (Supplementary Figure 2B). To better understand the molecular events that may lead to enhanced levels of hemoproteins, we detected the level of a putative heme sensor and heme chaperone necessary for maintenance of cellular heme and hemoprotein levels, PGRMC1 [44]. We found that PGRMC1 was elevated significantly 3 hours after treatment, but lowered to a level similar to those of untreated tumors 24 hours after treatment (Supplementary Figure 2C). Furthermore, we found that the level of mitochondrial structural protein Tom40 [45, 46] was not affected by CA4P treatment (Figure 6A). In contrast, the level of the mitochondrial fission protein Drp1 [34] was reduced at 3 and 24 hours post-treatment (Figure 6B), while the level of the mitochondrial fusion protein Mfn2 [35] was enhanced at 24 hours post-treatment (Figure 6C).

## DISCUSSION

CA4P is the leading VDA compound, inhibiting tubulin assembly into microtubules in tumor cells [36]. It causes vascular collapse, thereby reducing blood flow and supply of oxygen and nutrients [2]. Evidently, MSOT can detect changes in oxyhemoglobin and deoxyhemoglobin based on their unique absorptions. Consistent with previous studies of CA4P [8, 9, 14], MSOT showed that CA4P caused significant reduction of perfusion and oxygenation in tumors (Figures 1 & 2). Data from H&E staining and IHC (Figures 3 & 4) confirmed that CA4P caused vascular shutdown and induction of necrosis. Notably, these effects of CA4P are consistent with the results from another study of the effect of CA4P on chemically induced hepatocellular carcinomas (HCCs) in rats [47]. Using MRI and postmortem techniques, Liu and colleagues observed strong tumor necrosis in micro-HCCs, decreased blood perfusion in cirrhotic liver, and lowered tumor blood flow from intravascular to extravascular extracellular space [47].

Evidently, our data show that MSOT can be a new and useful tool for photoacoustic imaging to detect tumor hypoxia. MSOT uses endogenous hemoglobin as a contrasting agent, and it allows rapid and real-time monitoring of vascular oxygen dynamics in response to diverse therapies or treatment. Unlike other optical imaging methods, MSOT is unaffected by photon scattering and thus can provide high-resolution acoustic readout deep inside biological tissues [31]. The imaging of multiple absorption spectra is of major importance and differentiates MSOT from ultrasound imaging. It enables MSOT as a high-resolution molecular imaging modality, whereby not only structures but also specific intrinsic molecules or extrinsically administered agents can be uniquely identified based on their absorption spectra. Further, real–time optoacoustic measurements enable visualization of dynamic phenomena, such as the monitoring of physiological changes or contrast agent uptake and the minimization of motion artifacts [48, 49].

More importantly, by combining imaging and IHC techniques, we gained novel insights into the molecular mechanisms underlying tumor recurrence and treatment resistance to VDAs. While many clinical trials are ongoing to test the use of VDAs in cancer therapy, no VDAs have been approved for therapeutic purposes in patients to date [5, 50]. The main issue is that VDA treatment leads to the occurrence of a viable rim of tumor cells, which contributes to tumor regeneration, metastasis, and ongoing progression [20–23]. Likewise, CA4P leaves a rim of resistant tumor cells that may reperfuse and repopulate the tumor cells [36–38]. Although several mechanisms, such as the activation of tumor-associated microphages, have been proposed to explain tumor resistance to VDAs, these mechanisms cannot explain the entire phenomenon, and combination therapy based on these proposed mechanisms has not been proven effective in humans [20–23]. Importantly, our data here provide novel insights about how to improve the anti-cancer efficacy of VDAs.

Our data suggest that induced levels of heme-related proteins contribute to the occurrence of a viable rim of tumor cells in response to VDA treatment. Previously, we have shown that NSCLC cells exhibited substantially increased levels of an array of proteins promoting heme synthesis, uptake, and function [42, 43, 51]. Heme serves as an essential prosthetic group or cofactor for many proteins and enzymes involved in oxygen utilization and detoxification, such as mitochondrial respiratory chain complexes, catalases, and peroxidases, and promotes their expression [52, 53]. Heme is an essential metallonutrient for organisms ranging from bacteria to humans [43, 54–56]. As such, the increased levels of heme biosynthesis and uptake lead to intensified oxygen consumption and mitochondrial respiration, which fuel tumorigenic functions of NSCLC cells [42, 43, 51].

Our data here show that the levels of proteins involved in heme biosynthesis, uptake, and degradation, as well as oxygen-utilizing hemoproteins, are upregulated further in CA4P-treated resistant tumor cells (Figure 5 & Supplementary Figure 2). The elevation of PGRMC1, like ALAS1, at early time points likely contributes to the increase in hemoproteins. This increase would presumably further enhance the capability of resistant tumor cells to carry out oxidative phosphorylation and to combat ROS generated due to hypoxia. Additionally, the products of heme degradation, biliverdin and bilirubin, are potent antioxidants [57–59]. The elevated levels of HO-1 and increased heme availability in resistant tumor cells should lead to increased heme degradation products, which can further enhance the ability of cancer cells to combat oxidative stress and survive. Indeed, numerous previous studies support the pro-tumorigenic functions of HO-1 [58]. Through the actions of biliverdin and CO, HO-1 exhibits anti-oxidative and anti-inflammatory functions, respectively. Interestingly, another study showed that inhibition of heme degradation is synthetically lethal when combined with fumarate hydratase deficiency in hereditary leiomyomatosis and renal-cell cancer cells [60]. It was suggested that heme synthesis and degradation enable cells with fumarate hydratase deficiency to use the accumulated TCA cycle metabolites and permit partial mitochondrial NADH production. Clearly, multiple pathways can operate to promote the functions of HO-1 in carcinogenesis and tumor progression. In this report, our data show that heme flux and function are further elevated in CA4P-treated, resistant H1299 tumor cells, which have highly intensified heme biosynthesis and uptake compared to non-tumorigenic cells [41, 42].

It is also worth noting that markers of mitochondrial structure and function indicated that CA4P-treated resistant tumor cells are not undergoing mitochondrial fission and apoptosis. Recent studies have demonstrated that functional mitochondria are critical for tumorigenic function of many types of tumor cells, including NSCLC cells [42, 61, 62]. In CA4P-treated resistant tumor cells, the level of mitochondrial structural protein Tom40 was not affected, the level of Drp1 was reduced, and the level of Mfn2 was increased (Figure 6). Because increased levels of Mfn2 promote mitochondrial function and increased level of Drp1 is indicative of mitochondrial fragmentation and apoptosis, these results strongly suggest that CA4P treatment did not disrupt mitochondrial function in these cells [34, 35]. A previous study showed that CA4P decreases mitochondrial membrane potential and causes the release of proapoptotic mitochondrial membrane proteins in leukemic cells [63]. Here, we did not observe such an effect on viable lung tumor cells. This lack of effect is likely attributable to elevated heme and mitochondrial function in lung tumor cells which may render mitochondria more resistant to hypoxia [41–43]. Together, these results suggest that elevated proteins involved in heme flux and function are important mechanisms contributing to tumor reoccurrence and VDA-resistance. Furthermore, our data suggest that combination of VDAs with drugs that inhibit heme flux and function may be effective anti-cancer treatments. Hence, the results presented in this report have the potential to open up a new avenue of research in developing VDAs as effective drugs to combat lung cancer, as well as other aggressive cancers.

## MATERIALS AND METHODS

### Animals

The experiments were approved by the UT Southwestern Animal Care and Use Committee. Six to eight week old nude mice were obtained from Jackson Laboratories and maintained under a specific pathogen free environment with food and water provided *ad libitum*.

### Drug preparation

CA4P (Mateon Therapeutics) was prepared in saline and injected intraperitoneally (IP) at a dose of 120 mg/kg.

### *In vivo* bioluminescence imaging (BLI)

Female and male 8 week-old nude mice (each group n=6) were implanted with 3x10^6^ H1299-Luc NSCLC cells subcutaneously in the flank. After 3-4 weeks, when the tumor size reached 8-10 mm in diameter, bioluminescence images were taken with an IVIS Spectrum^®^ (Perkin-Elmer) at different time intervals (0h, 3h, and 24h). Mice were anesthetized in an isoflurane chamber (2% isoflurane and oxygen) and luciferin (sodium salt; Gold Biotechnology, St. Louis, MO; 60 μl of 40 mg/ml) was administered subcutaneously between the scapulae. A BLI time course was acquired over 30 mins (Exposure time: auto, F Stop: 2, binning: small) [9]. CA4P was administered IP (120 mg/kg in saline) after baseline BLI. 3 hours and 24 hours later, BLI scanning was repeated in the same setting with a new injection of luciferin.

### Multispectral optoacoustic tomography

H1299-Luc implanted nude mice were placed under anesthesia via inhalation of 2% isoflurane and air. The animal was transferred from the induction chamber to the animal holder. A thin layer of ultrasound gel was applied around the tumor region to provide optical and acoustic coupling to the membrane. In order to examine oxyhemoglobin and deoxyhemoglobin, mouse images were acquired in transaxial sections through the tumor region using seven wavelengths – 680, 715, 730, 768, 800, 850 and 900 nm – with an MSOT InVision 256-TF device (iThera Medical, Munich, Germany). Briefly, a tunable optical parametric oscillator (OPO) pumped by an Nd:YAG laser provided excitation pulses with a duration of 9 ns at wavelengths from 660 nm to 980 nm at a repetition rate of 10 Hz with a wavelength tuning speed of 10 ms and a peak pulse energy of 90 mJ at 720 nm. Ten arms of a fiber bundle provide uniform illumination of a ring-shaped light strip approximately 8 mm wide. For ultrasound detection, we used 256 toroidally focused ultrasound transducers with a center frequency of 5 MHz (60% bandwidth) organized in a concave array of 270 degree angular coverage and a radius of curvature of 4 cm. A model-based reconstruction was used prior to multispectral processing. Twenty frames per wavelength were acquired and averaged. Initially, the tumor region was imaged while the mice were breathing air. Then, the inspired gas was changed to 100% oxygen, and finally air again. To allow the animal to reach equilibrium with the different gas, the animal remained in the imaging chamber for five minutes without imaging. After five minutes, the tumor region was imaged using the same parameters. Images were reconstructed and processed using manufacturer’s software. Percentage of hemoglobin saturation (%SO_2_) was calculated as %SO_2_ = [HbO_2_/ (HbO_2_+Hb)]^*^100.

### Hematoxylin and eosin (H&E) staining

Following the final imaging, mice were sacrificed, tumors excised, and tumor tissue was prepared for histology. For H&E staining, tumor tissues (untreated control and 0h, 1h, 3h, and 24h after treatment with CA4P) were fixed in 4% formalin, embedded in paraffin and sectioned. Sections were 4 μm thick. Further, sections were stained with H&E. The extent of necrosis was estimated as the sum of all necrotic areas divided by total area using Zen software (Zeiss).

### Immunohistochemistry

Slides were deparaffinized with xylene (3×10 mins), hydrated through a graded series of ethanol solutions (100% 1×5 mins, 95% 1×5 mins, and 70% 1×2 mins), and washed with distilled water (1×2 mins) and 1X TBST (0.1 M TRIS-HCL, pH 7.5; 0.15 M NaCl; 0.05% Tween^®^20) for 1×2 mins. After antigen retrieval, samples were marked with a hydrophobic barrier pen (SPM0928, Fisher). Slides were blocked with 1X TBS (0.1 M TRIS-HCL, pH 7.5; 0.15 M NaCl) containing 10% goat serum (16210-072, Gibco). Primary antibodies were diluted in 1X TBS/1%BSA/10% goat serum and added drop-wise to slides to completely cover the tissue area. The dilutions were 1:200 for ALAS1 (sc-50531; Santa Cruz), 1:200 for HCP1 (sc-134997), 1:100 for HRG1 (sc-101957; Santa Cruz); 1:400 for HO-1 (sc-10789; Santa Cruz), 1:250 for Tom40 (sc-11414; Santa Cruz), 1:200 for Drp1 (sc-32898; Santa Cruz), 1:400 for Mfn2 (9482, Cell Signaling), 1:200 for cytochrome c (sc-7159, Santa Cruz), 1:200 for COX-2 (sc-7951, Santa Cruz), and 1:200 for PGRMC1 (13856, Cell Signaling). Sections were incubated with primary antibodies overnight at 4°C. Primary antibodies were aspirated and slides were washed 3×2 mins in 1X TBST. Slides were then incubated with horseradish peroxidase (HRP)-conjugated goat anti-rabbit IgG (31460; Thermo Scientific) at a dilution of 1:200 in 1X TBS/1%BSA for 45 mins at room temperature (RT) and washed 6×2 mins in 1X TBST. Slides were then incubated with tyramide signal amplification (TSA)-conjugated fluorophores, diluted 1:100 in 1X Plus Amplification Diluent, both obtained from the Opal 4-Color Manual IHC kit (NEL810001KT, PerkinElmer) for 10 mins at RT. TSA was aspirated, and slides were washed 3×2 mins in 1X TBST. DAPI, diluted in TBST, was added to slides and incubated for 5 min at RT, followed by 3×2 mins washes in 1X TBST. Coverslips were mounted over the slides using VECTASHIELD mounting medium for fluorescence (Vector Laboratories), sealed with nail polish, and stored at −20°C in the dark.

Slides were scanned at a 40× resolution with an Olympus VS120 slide scanner with the VS-ASW-L100 software. DAPI was used to visualize nuclei and therefore differentiate viable regions from necrotic regions, which can be detected in H&E stains as well. Quantification of signal intensity was performed with the cellSens Dimension software following the manufacturer’s instructions (Olympus). 7-10 regions of interest (ROIs) of equal area were identified in the viable tumor regions. The ROIs were positioned evenly throughout the tumor tissue and outside of the areas of necrosis and artifacts. ROIs were retested under three different filters, FITC, Cy3, and Cy5, to ensure that no artifacts were present. ROIs were re-positioned if artifacts were present under one or more filters. The thresholds were set so that the image noise disappeared and only the cells were visible. Signal intensities of ROIs were acquired and averaged. The mean signal intensity for each antigen was obtained by subtracting the corresponding negative control average (signal obtained from tumor tissues without the primary antibody) from the signal average with the primary antibody. Results from all antibodies were repeated at least twice.

## SUPPLEMENTARY MATERIALS FIGURES


