# Supplementary material for: The vascular disrupting agent combretastatin A-4 phosphate causes prolonged elevation of proteins involved in heme flux and function in resistant tumor cells
Source: Oncotarget. 2017 Dec 28;9(3):4090–101. doi: 10.18632/oncotarget.23734 (PMC5790523; doi:10.18632/oncotarget.23734)
Supplement: Supplementary file 1 [file oncotarget-09-4090-s001.pdf]

The vascular disrupting agent combretastatin A-4 phosphate causes prolonged elevation of proteins involved in heme flux and function in resistant tumor cells

SUPPLEMENTARY FIGURES

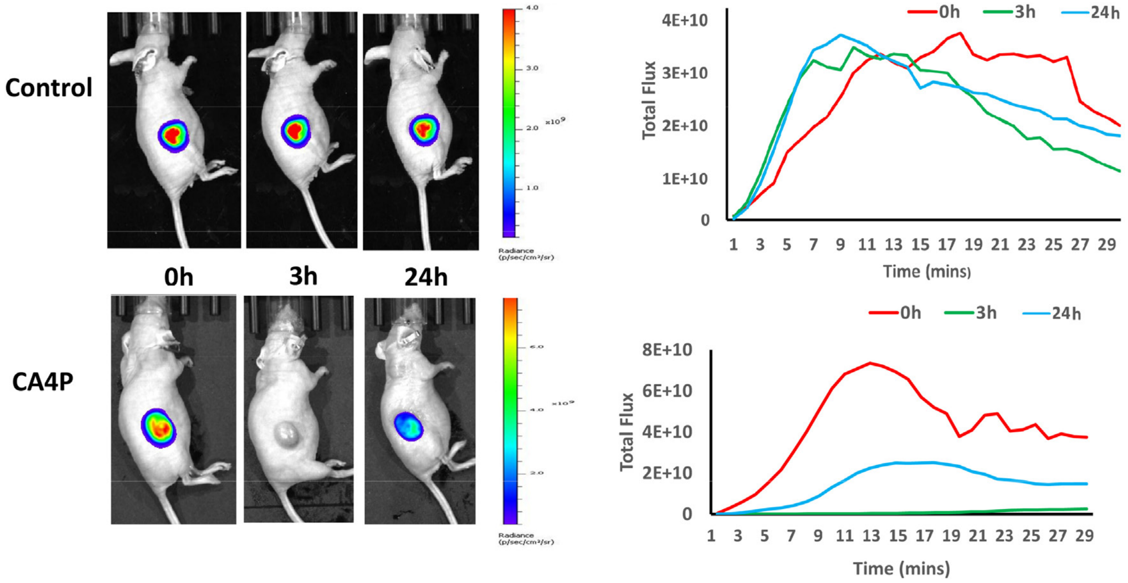

Supplementary Figure 1: Dynamic BLI, following subcutaneous administration of luciferin on each occasion, showing tumor response at different time points (0h, 3h, and 24h) post-saline (control) or CA4P administration (120 mg/kg, IP). Note that the tumor vasculature was shut down 3 hours after CA4P administration, and therefore, the substrate luciferin could not reach the tumor, causing the absence of luminescence signals.

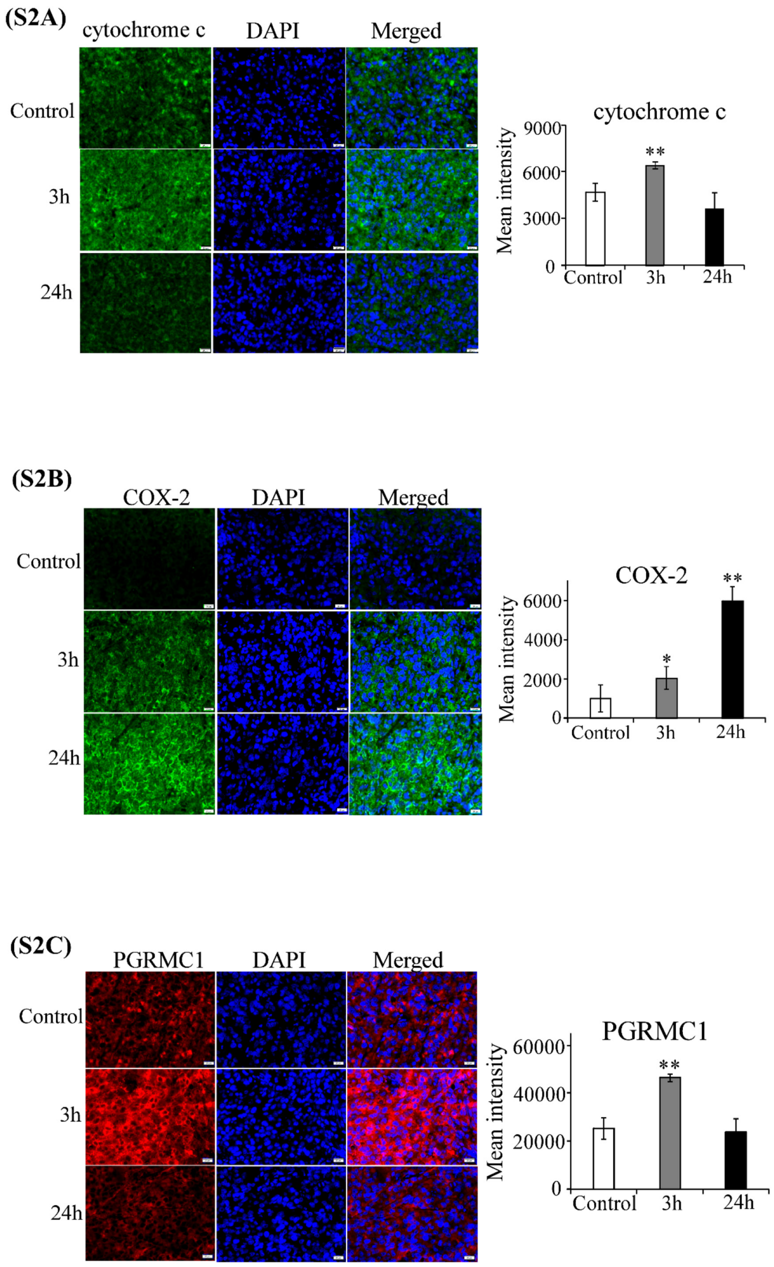

Supplementary Figure 2: CA4P affects expression of hemoproteins and putative heme sensor. Representative images of CA4P-treated, fluorescent immunohistochemically stained paraffin sections of subcutaneous xenograft tumors including untreated control (Row 1), 3 hours post-CA4P (Row 2), and 24 hours post-CA4P (Row 3). Bar graphs indicate the quantification of mean grey intensity. Data are presented as mean ± SEM (n=10, *p value < 0.05; **p value < 0.005; scale bar, 20 μm). **(A)** cytochrome c is significantly elevated 3 hours after treatment. At 24 hours post-treatment, it is not significantly different from untreated tumors. Anti-cytochrome c antibody (green), DAPI for nuclei (blue), and merged image (green and blue). **(B)** COX-2 is significantly enhanced at both 3 and 24 hours post-treatment, with higher expression at 24 hours post-treatment. Anti-COX-2 antibody (green), DAPI for nuclei (blue), and merged image (green and blue). **(C)** PGRMC1 is elevated significantly at 3 hours post-treatment. PGRMC1 level are similar to those of untreated tumors at 24 hours post-treatment. Anti-PGRMC1 antibody (red), DAPI for nuclei (blue), and merged image (red and blue).
